# Efficient Object Detection Based on Masking Semantic Segmentation Region for Lightweight Embedded Processors

**DOI:** 10.3390/s22228890

**Published:** 2022-11-17

**Authors:** Heuijee Yun, Daejin Park

**Affiliations:** School of Electronic and Electrical Engineering, Kyungpook National University, Daegu 41566, Republic of Korea

**Keywords:** autonomous driving, object detection, OpenCV, ENet, YOLO, deep learning

## Abstract

Because of the development of image processing using cameras and the subsequent development of artificial intelligence technology, various fields have begun to develop. However, it is difficult to implement an image processing algorithm that requires a lot of calculations on a light board. This paper proposes a method using real-time deep learning object recognition algorithms in lightweight embedded boards. We have developed an algorithm suitable for lightweight embedded boards by appropriately using two deep neural network architectures. The first architecture requires small computational volumes, although it provides low accuracy. The second architecture uses large computational volumes and provides high accuracy. The area is determined using the first architecture, which processes semantic segmentation with relatively little computation. After masking the area using the more accurate deep learning architecture, object detection is implemented with improved accuracy, as the image is filtered by segmentation and the cases that have not been recognized by various variables, such as differentiation from the background, are excluded. OpenCV (Open source Computer Vision) is used to process input images in Python, and images are processed using an efficient neural network (ENet) and You Only Look Once (YOLO). By running this algorithm, the average error can be reduced by approximately 2.4 times, allowing for more accurate object detection. In addition, object recognition can be performed in real time for lightweight embedded boards, as a rate of about 4 FPS (frames per second) is achieved.

## 1. Introduction

Currently, with the advancement of artificial intelligence technology, industries in various fields, ranging from automobiles to the Internet of Things (IoT), are developing. In these industries, artificial intelligence calculates the input of multiple datasets and converts it into the required output data [1,2]. Various types of sensors are used to receive data, among which camera sensors and methods for processing visual information input are active fields of research [3,4]. Object recognition using visual data as learning data for deep learning is used in various methods and has been researched in a variety of fields [5]. However, these data are difficult to process in real time using a processor that has small amount of memory because of the large amount of image data. In addition, to implement artificial intelligence in daily life a lightweight embedded board must be used. However, lightweight embedded boards are not suitable for large computation loads, as they have small memory and power.

The weight reduction of the object recognition algorithm using a camera sensor has always been an important task to be solved, and research is currently being conducted in various ways [6]. Various methods of processing images have been developed for effective implement of algorithms [7]. However, as with all algorithms, there is a trade-off relationship between accuracy, speed, cost, and amount of computation. The ASM framework has been studied as an effective method for mining most unlabeled or partially labeled data to enhance object detection. The ASM framework can be used to build effective CNN detectors that require fewer labeled training instances while achieving promising results [8].

**Figure 1:**
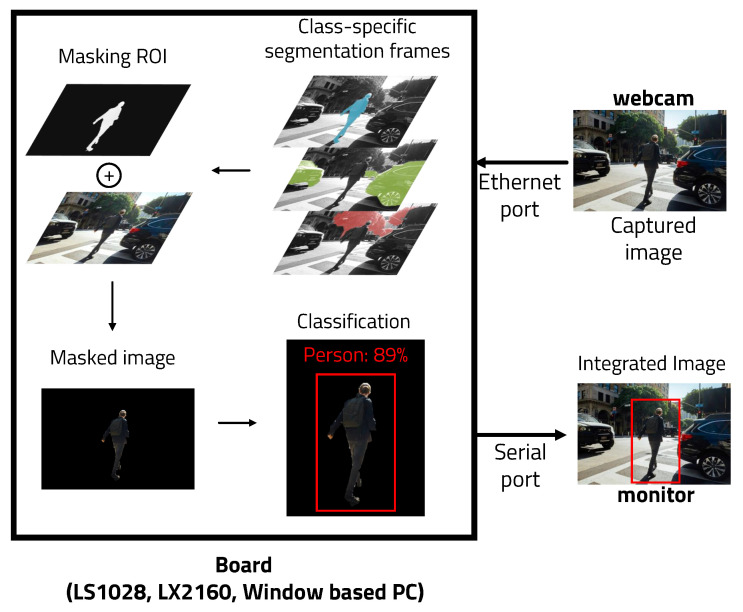
Overall structure.

This paper introduces an object recognition algorithm based on deep learning to accurately recognize objects in real time. YOLO (You Look Only Once), a deep learning-based object recognition architecture, is currently the most well-known and efficient object recognition algorithm. However, it is too heavy an architecture to use in real-time on a lightweight embedded board. Therefore, the ROI (Region of Interest) is set in the input data to reduce the amount of image processing. Figure 1 shows the overall operation of the algorithm. The ROI can be set using ENet (Efficient Neural network), a semantic segmentation architecture based on deep learning. The the object of interest can be expressed in a specific color using semantic segmentation. By binarizing this expression, the remaining parts other than the recognized object are removed. Because this architecture only recognizes people, it is useful for removing objects other than people. Running YOLO using masked images as input data reduces computation and can be used on a lightweight embedded board, resulting in improved accuracy.

By dividing image processing into two steps in this way, the efficiency can be maximized, and the accuracy does not change rapidly in various environments. By setting the ROI after filtering using segments in the input image, cases that were not recognized by different variables, such as differentiation from the background, can be excluded, increasing the accuracy. Using two deep learning models allows for implementation with higher accuracy and faster execution time. When the amount of computation is reduced and the algorithm is implemented on a lightweight embedded board, its scope of use can be widened considerably.

## 2. Background

### 2.1. Preliminary Study of the Proposed Method

Deep learning is a technology that trains rules from data using artificial neural networks. An artificial neural network composed of a layer of neurons is first trained with sample data before being used to make inferences. Deep learning is trained with the help of a deep artificial neural network composed of multiple layers. As the data pass through a series of filters in a deep neural network, they can be usefully refined to handle multi-step information extraction. When analyzing images using deep learning, images are first classified. After dividing the image into defective and regular components, they are sorted and assigned by class. The image is then subdivided and each pixel is assigned to a class before image processing is performed. A significant amount of computation is required to realize the repeatability of deep learning algorithms, added complexity as the number of neural network layers increases, and implementation of the data required for training.

R-CNN (Regions with Convolutional Neuron Networks features) [9], an existing image processing system, creates a bounding box on an object by a method called region proposal, then applies a classifier to the box to classify it. After classification, it proceeds through a complex process of adjusting the boxes and removing duplicate detections. The image is then post-processed to estimate the detection probability of the boxes. Because these processes need to be trained and optimized independently, the overhead is large, requiring a significant amount of processing time.

**Figure 2:**
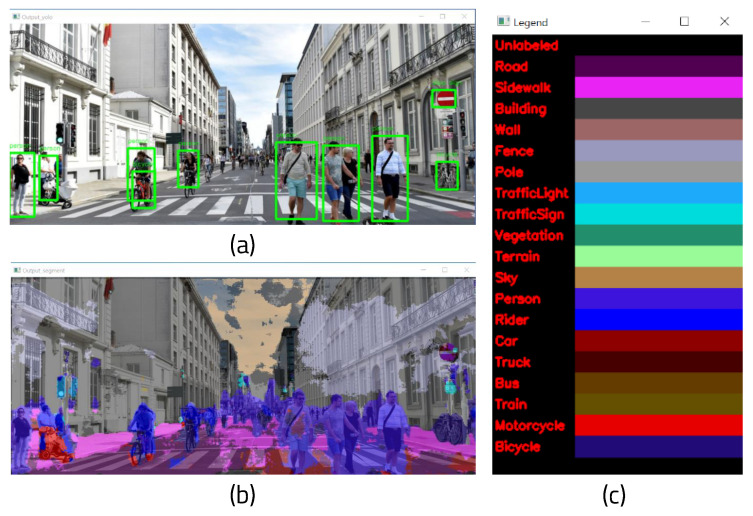
(**a**) Result of YOLO object detection, (**b**) result of semantic segmentation, and (**c**) classes and labels of semantic segmentation.

YOLO (You Look Only Once) [10] is a real-time image detection architecture based on deep learning. Unlike RCNN, YOLO processes images in a single regression without requiring multiple steps. Using one pipeline, it can detect a target object by looking at the image once. It finds the coordinate position and probability of the bounding box in the image pixel. In addition, because the entire image is viewed and processed, it does not recognize background noise as an object, and as such the background error is small. Fast YOLO, which consists of a total of 24 convolution layers and two fully connected layers, has nine convolutional layers. YOLO convolution layers can be trained with datasets. YOLO uses a framework called darknet to enable training and inference. Figure 2a shows a box drawn around the recognized object, with its class and probability shown in the kernel.

Semantic segmentation [11] classifies all pixels in an image into a designated class to recognize objects. It is currently used in various fields, including self-driving cars and medical image analysis. The difference between SS and object recognition algorithms such as YOLO is that the former determine which class the pixels themselves belong to. As a result, the number of people or objects in the image cannot be counted; only the types of recognized objects can be identified.

There are numerous algorithm models for implementing semantic segmentation, of which the fully convolutional network (FCN) approach [12] is the most well-known. Because an FCN only consists of convolutional layers, it is not necessary to fix the size of the input image. In addition, because the filter is learnable and stems from a single deep learning model, an end-to-end model can be used. Because it processes the entire image at once, it can be processed quickly. However, it has the disadvantage of lower the accuracy when the resolution changes during the processing process. ENet (Efficient Neural Network) [13] is a deep learning-based semantic segmentation structure that uses an algorithm model called ResNet [14]. Although the existing method requires a large amount of computation, consumes a lot of power, and has a slow processing speed when classifying object classes in units of pixels, ENet has developed a new deep neural network structure that can be performed on an embedded board. ENet is made up of an encoder and a decoder along with thirteen convolution filters. A sampling operation is used to solve the problem of resolution loss and increase the accuracy. As shown in Figure 2b,c, objects within the image can be classified using the color assigned to each class.

### 2.2. Concepts and Definitions of the Proposed Method

The method devised in this paper involves the design of a more lightweight object recognition algorithm using the two deep learning models described above. A filter is created for object recognition in the image. This filter recognizes the pixels an object occupies in the image, and masks only the object. The more complex the background excluding the object in the image, the lower the accuracy and the greater the amount of computation. Therefore, it is possible to reduce the amount of computation by recognizing the filtered image as an object.

This study conducted further experiments based on previous studies [15]. In the mentioned study, the authors used two lightweight embedded boards and measured for memory and time as well as for various elements such as background complexity and power consumption. They were able to identify efficiency and accuracy by experimenting with various versions of YOLO that recognize objects.

There have been other studies using YOLO by setting ROI as segmentation. In [16], the authors used YOLO to set ROI by segmentation and then executed object recognition with CNN. Similar to the present study, they implemented it in two stages. The structure was used for interpreting sign language with deep learning. After extracting hand parts using YOLO, sign language was interpreted by a CNN. When learning by setting ROI, it is apparent that large amounts of data are not needed and that the speed and success rate are improved.

## 3. Implementation

**Figure 3:**
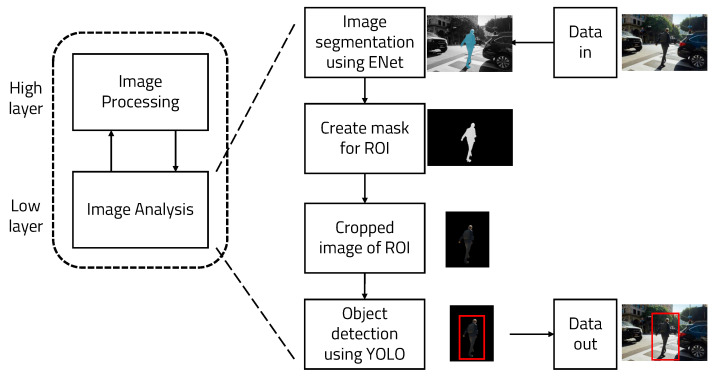
Overall algorithm used for real-time object detection.

We propose a method to execute the object recognition algorithm in real time on a lightweight embedded board by writing a lightweight and divisional-sized algorithm. Two deep learning-based systems were used to reduce the amount of computation and increase the accuracy and overall structure, as can be seen in Figure 3.

The reason why a model that integrated segmentation and object detection was not used from the beginning was to divide the code. There is benefit in separating segmentation and object detection. First, ENet and pre-image postprocessing can be operated on a very small FPGA board, and YOLO can be run on a better performance FPGA. Combining segmentation and detection into one model requires an embedded board that performs much better than is necessary when running each individually. Two boards with relatively poor performance are much more cost-effective than one high-performance board. In addition, the communication time between the two FPGA boards is very short (less than 0.1 s), and the communication cost is insignificant because it uses less energy, which is more effective.

### 3.1. Semantic Segmentation Object Detection

**Figure 4:**
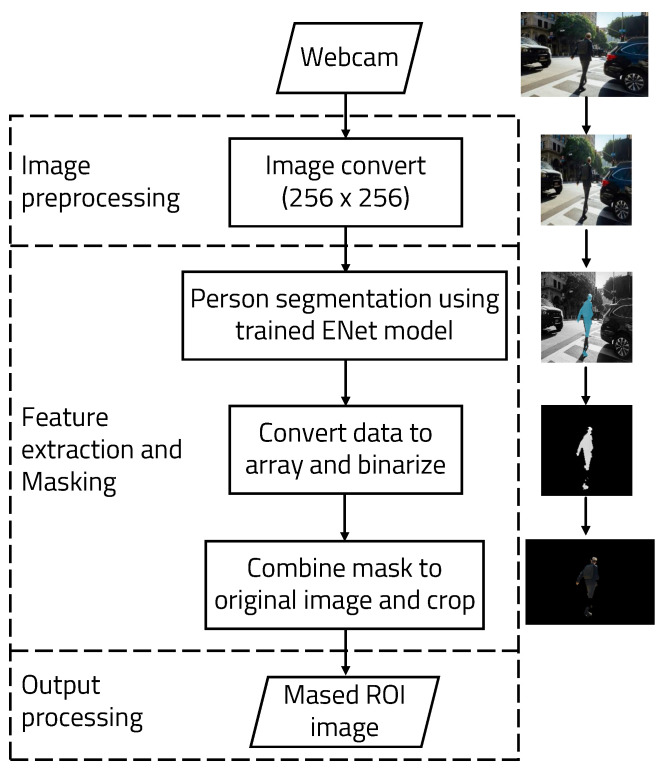
Algorithm used for semantic segmentation.

Figure 4 shows the diagram of the semantic segmentation algorithm using ENet. The video data received by the webcam are captured as frame units. One frame is captured every 0.03 s on average. The frame is masked using the trained weights. Using trained weights, a human in an image can be detected and segmented. This allows the ROI to be selected for object recognition in the image. We used ENet because it is easily trained to detect specific targets and has higher accuracy. When training ENet, the UP-S31 dataset from the Leeds Sports Pose dataset with the MPII Human Pose Dataset was used to recognize humans [17].

Currently, with the rapid development of deep learning and computer vision fields, many highly diverse models are being developed, such as DUC-HDC (Dense Upsampling Convolution and Hybrid Dilated Convolution) and DCNAS (Densely Connected Neural Architecture Search). However, it was difficult to find a model that could be be executed simultaneously in different in operating systems, taking into account the differences in the board and the host PC and their corresponding execution methods. Pytorch, Mask-RCNN, and ENet, were judged to be the most stable and widely known for the two operating systems. Thus, these were the main models tested, and the study was conducted according to the results.

**Figure 5:**
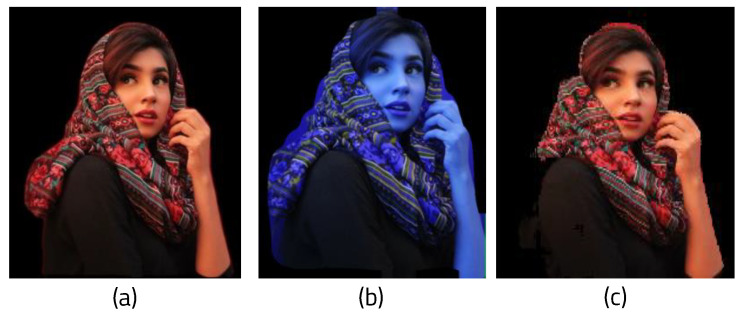
Results of segmentation algorithms: (**a**) Pytorch, (**b**) Mask RCNN, (**c**) ENet.

**Figure 6:**
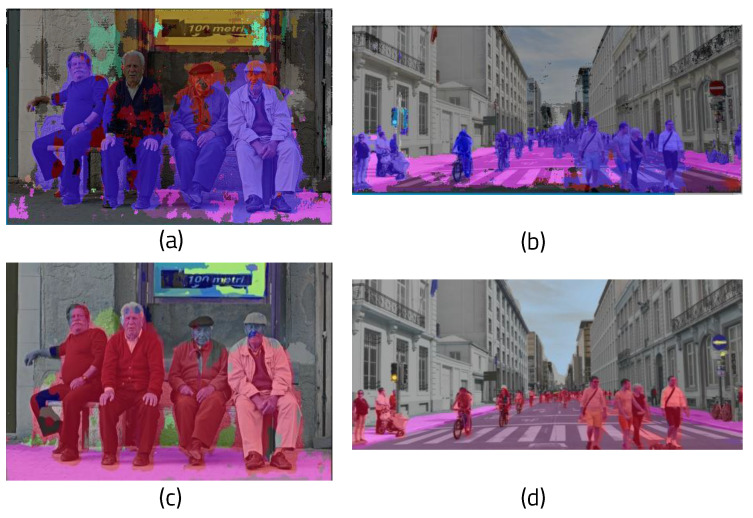
Results for IC-Net and ENet: (**a**,**b**) results for ENet; (**c**,**d**) results for IC-Net.

We tested with pytorch, mask RCNN, and ENet. For a single frame, ENet took 1.75 s, Pytorch took 4.45 s, and Mask RCNN took 1.22 s. We tested these models on the same PC based on Windows 10. The input image was 640 × 959 in size and was a jpeg file. In order to check whether segmentation was performed well, an image containing only one person was tested. The accuracy was 0.89 for ENet, 0.92 for Pytorch, and 0.84 for Mask RCNN. Figure 5 shows the results with each algorithm. Eventually, ENet was chosen, as it was most efficient in terms of time and accuracy. Another model, IC-Net, [18] was tested as well. The time and accuracy of IC-Net were calculated using the same Cityscapes dataset. The average FPS (frames per second) rate is the time taken per frame, and can be calculated by dividing the total time taken to process the image by one. The average FPS of IC-Net on the host PC was 1.196, and the average FPS rate on the LS1028 board was 0.1402. Accuracy can be calculated by dividing the total number of people recognized after image processing by the number of people actually in the image. The accuracy was calculated as 0.73. Figure 6 shows the results with the different frameworks segmentation algorithm when using weights trained with the same Cityscape dataset; (a,b) show the results for ENet, and (c,d) show the results for IC-Net. Although the accuracy of the two models is similar, there are many differences in terms of FPS. Although the accuracy of segmentation is low, because image processing is performed again after ROI setting it is not necessary to sacrifice power or memory consumption for higher FPS and accuracy.

Algorithm 1 shows the semantic segmentation algorithm in more detail in the form of pseudo-code. This algorithm makes it easy to process the image received from the webcam by dividing it into frames utilizing OpenCV (Open-Source Computer Vision) functions. A trained model used in ENet is loaded using Keras and Tensorflow [19,20]. Because the weights trained in ENet process images with a size of 256 × 256, the initial width of the image should be 256. To obtain the memory usage within a Python program, the psutil Python package was used to create a function that outputs the memory in MB.
**Algorithm 1:** Pseudo-code of semantic segmentation algorithm
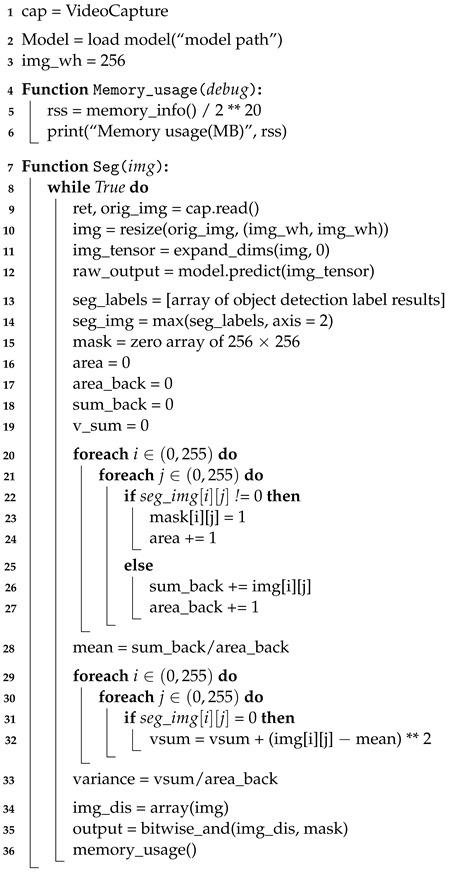


**Figure 7:**
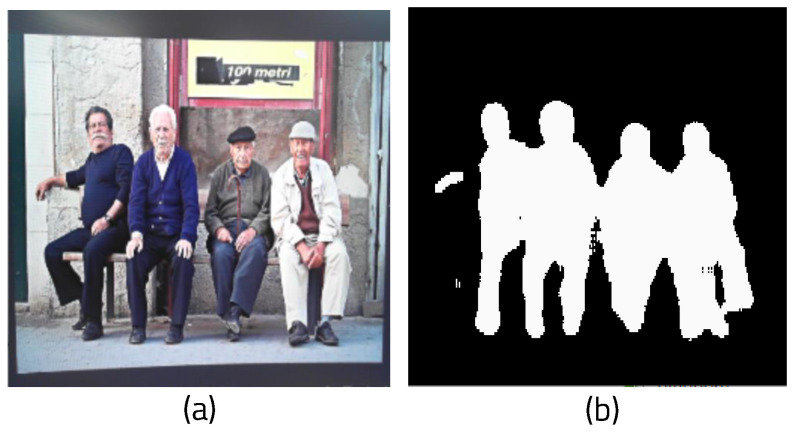
Results of masking: (**a**) original frame and (**b**) masked image.

We created a function that receives the frame image from the webcam as input and performs segmentation. It first receives the original frame size, then changes the webcam frame size (which was 640 × 480) to 256 × 256, the width of the initialized image. The batch dimension can then be set using the NumPY function. ENet loads a model trained to recognize only humans and runs it. Because the segmentation result is colored based on the recognized person, only the colored part needs to be extracted for masking. First, the labeled part of the result is converted into an array and combined with the image array. Before masking, the mask array and variables are initialized to calculate the amount of computation based on the complexity of the background. If the array of the segmented result image for masking is not 0, the mask array is set to 1 in order to binarize it. Figure 7a shows the original image, and Figure 7b shows the binarized mask showing only the part of the image recognized as a person.

**Figure 8:**
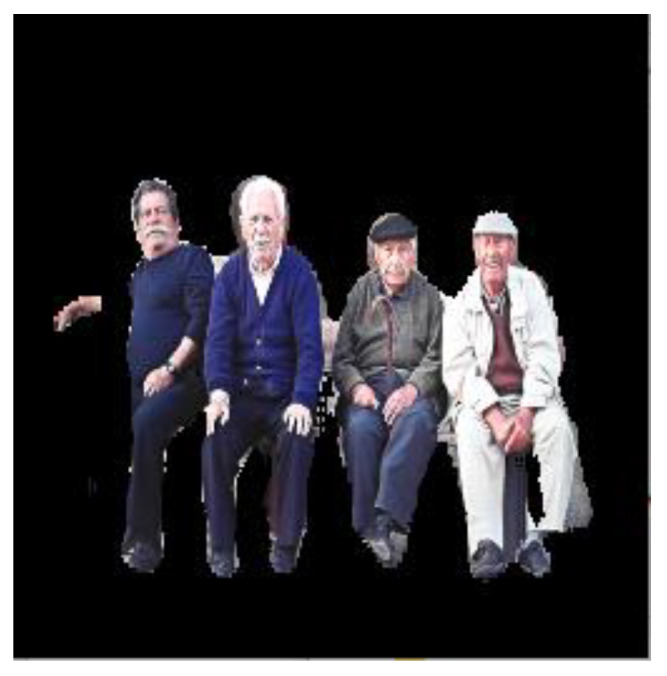
Results of ROI masked image.

In addition, the area variable is set to 1 whenever a pixel occupied by an object is found in order to determine how much space the recognized object takes up in the background. This helps to measure how image background complexity affects the performance of the algorithm. If the data of the segmented pixel is zero, it means that the pixel has no data on the segmented object. Therefore, it can be judged as the background of the image. To calculate the complexity of the background, the pixel value of the original image is added to obtain the background variance, then 1 is added to area_back. To determine the variance of the RGB values of the background, first, the average value of the background is obtained, then the sum of the deviations is computed. The variance can be calculated by dividing the sum of the deviations by the total number of elements in the entire background. To create a masked image, the original image is converted into an array, then the mask and the original image are combined using the bitwise and operation functions in OpenCV. Figure 8 shows the masking result by combining the original image and the mask. The memory usage function that was previously created is called to determine the computational cost of this process.

### 3.2. Object Detection with YOLO

**Figure 9:**
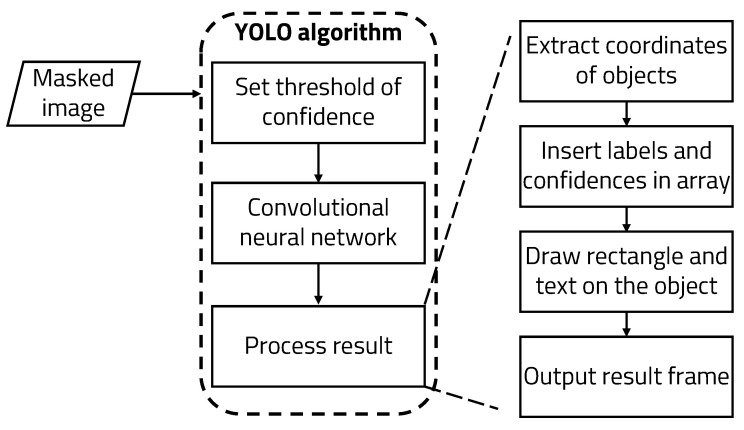
YOLO algorithm for object detection.

Figure 9 shows the structure of the YOLO algorithm, which recognizes an object in an image that was previously masked using ROI with segmentation. The YOLO model recognizes the masked image, and the box and probability of the recognized object are displayed.

**Figure 10:**
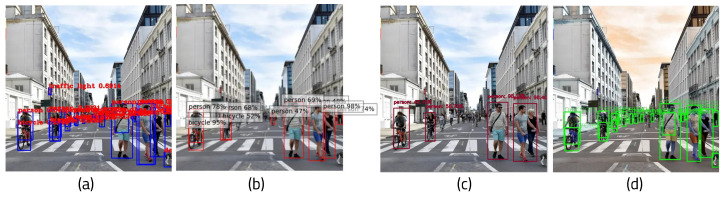
Results for stage one target detection algorithms: (**a**) YOLO, (**b**) SSD, (**c**) MobileNet, (**d**) Faster R-CNN.

As a first stage target, detection algorithms are implemented in a way that is lightweight and can be operated quickly and accurately; two algorithms, YOLO, and SSD, were tested [21] while running region proposal and classification simultaneously. It is not necessary to use a neural network called RPN (Region Proposal Network) to generate candidate targets. These algorithms predict target locations and classes directly over the network. As it is a method for solving the classification and localization problems at the same time, it can be simulated in high FPS. An efficient deep neural network model called MobileNets was tested as well [22]. MobileNet is an efficient convolutional neural network for low power devices. MobileNet utilizes depth-wise separable convolution to make the model lightweight. Two parameters were used to optimally fit MobileNet in memory-constrained environments. These two parameters adjust the balance between latency and accuracy. For better variety in the experiment, Faster R-CNN [23], a two stage detector, was tested as well. In a two-stage detector, regional proposals and classification are performed sequentially. The R-CNN algorithm has a limitation in that it is slower than YOLO. Fast R-CNN greatly reduces iterative CNN computations; however, the region proposal algorithm becomes the bottleneck. Faster R-CNN uses an RPN (Region Proposal Network) in the region proposal process to make the existing Fast R-CNN faster. Figure 10 shows the results of three algorithms. They were tested on the same PC based on Windows 10, and the weights were all trained using the Pascal VOC dataset. For simple detection, the input image was converted to 512 × 512. SDD with Pytorch took 7.6 s, YOLO took 4.24 s, MobileNet took 0.09 s, and Faster R-CNN took 7.3 s. The run time of the MobileNet model was overwhelmingly short, while the accuracy between YOLO and Faster R-CNN was quite similar. However, for the second stage of object detection, it is necessary to operate with high accuracy. As can be seen from the results, YOLO is the most accurate algorithm, and is fast as well. Eventually, YOLO was chosen for the second stage of object detection for subsequent experiments.

Algorithm 2 shows the YOLO object recognition algorithm in pseudo-code. YOLO is an algorithm developed on a C++-based framework called darknet in Linux. However, in order to use Tensorflow and various Python packages, darkflow [24], which ports YOLO to other Tensorflow-based platforms, was used. It is convenient because the number of codes is reduced and multiple functions can be used. In addition, it is faster than darknet. Using darkflow in Tiny-YOLO weight executes in 1.3 fps, while darknet takes 0.3 fps [25]. To run darkflow, the model’s path and weights are first set to be executed in YOLO. We used the YOLOv3 and Tiny-YOLOv3 weights, as they are stabilized for darkflow. YOLO is being developed in various ways, such as YOLOv5 and PP-YOLO. However, there were problems in the process of building and running it on the board, which are currently being resolved. We plan to present a more advanced architecture in the future. Even if the confidence threshold is set low, the accuracy hardly changes when setting the ROI. The TFNet class object was initialized with the previously set values to build the model. Random numbers were generated to determine the color to be used when displaying the recognized object.

By receiving an image with ROI masked by segmentation as input, the image can be analyzed with the previously built model using TFNet. The current time is recorded in a variable called start_time to calculate the processing time and fps. This initializes obj_count, a variable that counts the number of people to calculate accuracy. In order to show the result of the analyzed image, the coordinates of the upper part of the recognized object are stored as the tl variable, and the coordinates of the lower part of the object are stored as the br variable. The class and probability of the recognized object are stored as the text variable, so called to make it easily printed into the image.
**Algorithm 2:** Pseudo-code for YOLO object detection.
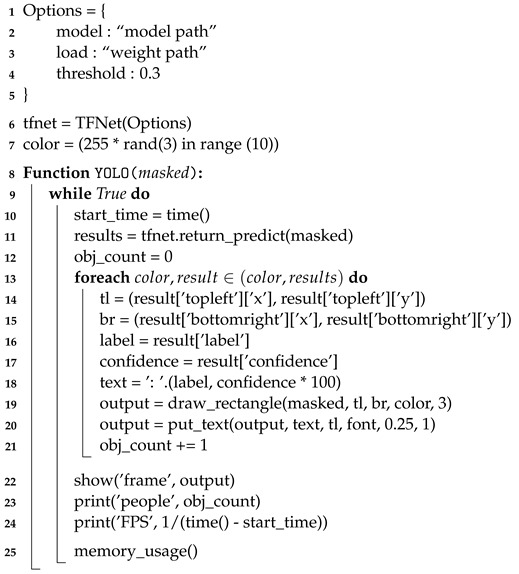


**Figure 11:**
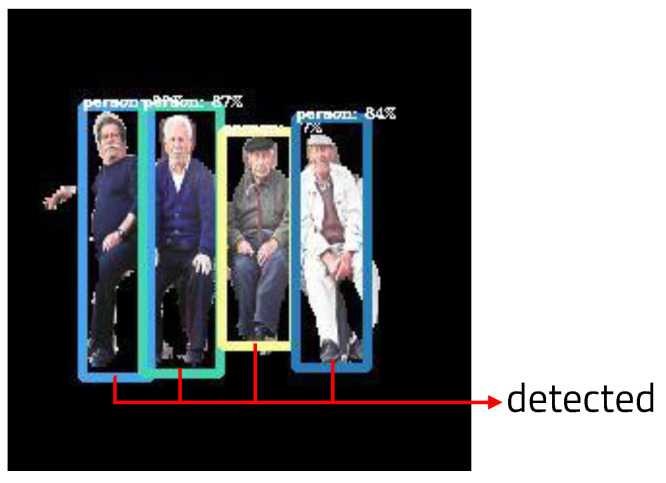
Results of YOLO object detection.

A box is drawn on the recognized object using the values stored in the variables tl and bl; then, the class and probability of the object are stated in the upper part of the box. Each time a box is drawn, one object is added to obj_count to count the number of recognized objects. In order to display the result again in real-time, the output is displayed using the OpenCV function. Figure 11 shows the resulting image recognized as only humans using YOLO in the ROI set by semantic segmentation. The number of people recognized and the fps can be calculated and printed. Using the memory usage calculation function written in the semantic segmentation algorithm, the amount of computation when processing images using YOLO can be calculated.

## 4. Experimental Setup and Evaluation

This section describes the image classification neural network target program and evaluates the proposed fault detector performance with random test images and fault injections.

### 4.1. Experimental Environment

**Figure 12:**
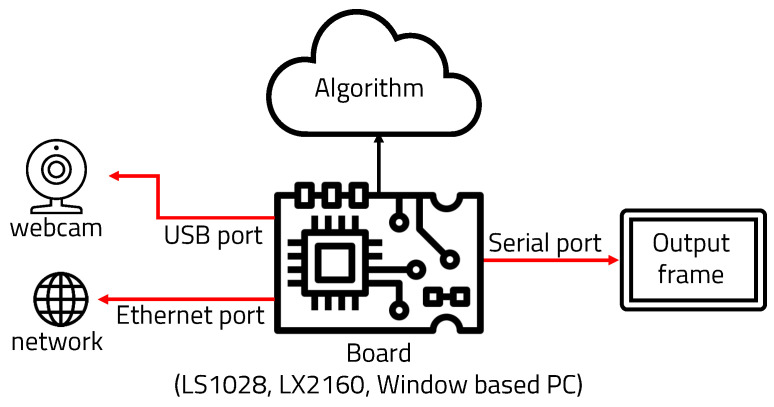
Structure of boards and algorithms.

Experiments with object recognition algorithms based on semantic segmentation and YOLO were conducted on the LS1028a [26] and LX2160a [27] boards as well as a Windows 10 PC. The boards were configured as shown in Figure 12. In order to determine whether the algorithms could be run on multiple operating systems, they were run on a Linux-based board and a Windows-based board for the experiments. Because these two operating systems have different methods of executing programs, memory, and performance on each board, various results were obtained. The LS1028a and LS2160a are processors made by the NXP company. The LS1028ardb is equipped with two 64-bit ARM Cortex-A72 processors with a maximum operating speed of 1.3 GHz per core, while the LX2160a is equipped with sixteen 64-bit ARMv8 Cortex-A72 processors, and the maximum operating speed per core is 2.2 GHz. They can both manage input/output and communication through an ethernet port, USB port and serial port. Python code was used for measuring execution time, memory usage, FPS, and number of detected people. To measure power consumption, Open Hardware Monitor was for host PC (Windows) and the Powertop tool was used for the Linux FPGA board.

### 4.2. Execution Time and Accuracy

#### 4.2.1. Methods for Measuring the Accuracy of Deep Learning Models

There are several methods for measuring the accuracy of a deep learning model; here, we used a confusion matrix [28]. The confusion matrix can be divided into four states: TP, when the model corrects the correct answer; TN, when the model predicts the correct answer incorrectly; FP, when the model incorrectly predicts the correct answer as an incorrect answer; and FN, when the model incorrectly predicts the incorrect answer as an incorrect answer. Equation (1) is the formula for calculating accuracy:

(1) $$\mathrm{Accuracy}=\frac{\mathrm{TP}+\mathrm{TN}}{\mathrm{TP}+\mathrm{TN}+\mathrm{FP}+\mathrm{FN}}.$$

In addition, precision, recall, and F1-score were calculated for various performance evaluation certificates. Precision was calculated using Equation (2), recall was calculated with Equation (3), and F1-score was calculated with Equation (4). The precision, recall, and the following formulas were used to calculate F1-score. We calculated TP as the number of people detected by YOLO, FP as the number of detected objects that were not human, and FN as the number of people that were not detected.

(2) $$\mathrm{Precision}=\frac{\mathrm{TP}}{\mathrm{TP}+\mathrm{FP}},$$

and

(3) $$\mathrm{Recall}=\frac{\mathrm{TP}}{\mathrm{TP}+\mathrm{FN}},$$

and

(4) $$F1-\mathrm{score}=\frac{2}{\frac{1}{\mathrm{precision}}+\frac{1}{\mathrm{recall}}}.$$

#### 4.2.2. Time and Measurement Accuracy Results of the Proposed Algorithm

**Figure 13:**
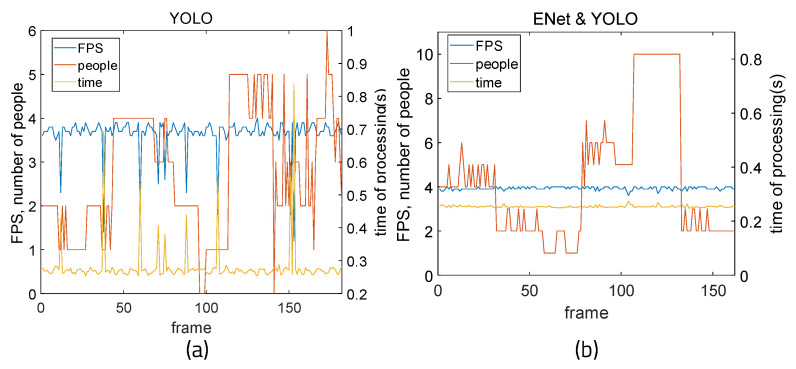
Time measurement: (**a**) YOLO and (**b**) YOLO executed with ENet.

**Figure 14:**
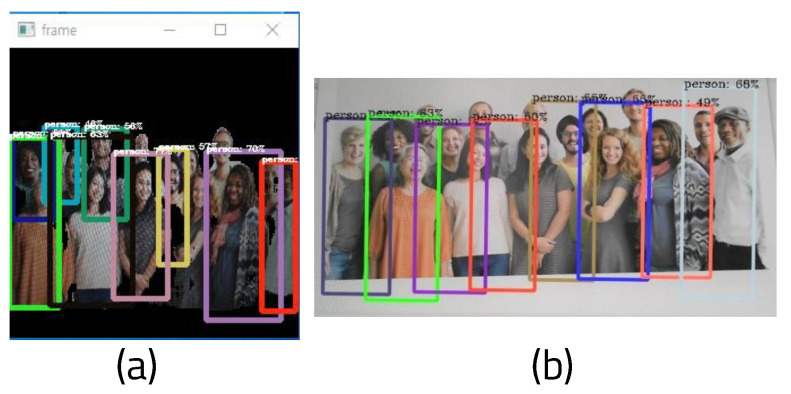
Result of detection of numerous people: (**a**) YOLO executed with ENet and (**b**) YOLO.

Figure 13 shows the results of measurements of time, fps, and the number of people detected when running only YOLO and when running YOLO and ENet on a Windows 10-based PC. Because there is a limit when measuring places with a large change in the number of people in real time, this was experimented with by showing pictures including different people on a webcam. Changes were measured in terms of FPS, power consumption, and memory usage while turning seventeen pictures containing different numbers of people into a slide show with a real-time webcam, which is demonstrated in Figure 14. When YOLO is used alone, fps and processing time values fluctuate according to the changing number of people. This means that if the floating population is high, operating YOLO alone is not be stable. The dispersion of fps can be derived by calculating the average fps and subtracting each fps value from the average, then adding all differences from the mean and dividing by the number of fps values. Here, the average fps is 3.64 and the dispersion of fps is 0.166. However, it can be seen that the fps and processing time are stable regardless of the changing number of people when integrating ENet and YOLO, as the average fps is 3.93 and the dispersion of fps is 0.0051.

**Figure 15:**
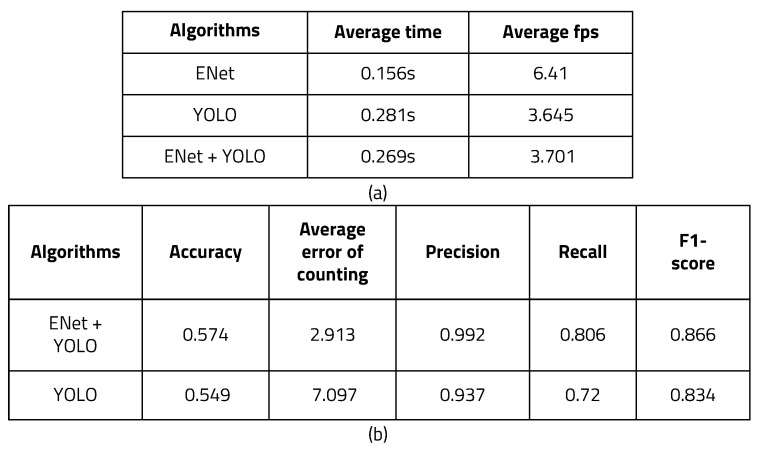
Time and accuracy measurement: (**a**) time and FPS measurement and (**b**) accuracy measurement.

Figure 15 shows the measurement results of average time, fps, accuracy, and error when using YOLO alone and when using ENet and YOLO together running on a Windows 10-based PC. With far fewer convolutional layers than YOLO and a fast and compact encoder–decoder structure, ENet’s average processing time was 0.156 s per frame, and the average fps was 6.41. When only YOLO was used, the average processing time per frame was 0.281 s and the average fps was 3.645 s. When ENet results were processed with YOLO, the average time was 0.269 s and the average fps was 3.701.

As can be seen, the method of setting ROI using ENet and processing it as YOLO input can reduce YOLO’s execution burden as a result of lowering the threshold of YOLO. The total number of recognition frames was divided by the number of people in the image and the number of frames correctly recognized to determine the accuracy of the object recognition algorithms. A clear improvement in accuracy can be seen when using YOLO and ENet together. Because TP and TN accurately calculated the part where the number of people matched, the error was calculated to obtain more appropriate accuracy by comparing the recognized number of people and the actual number of people. When only YOLO was used, the average recognition error was 7.097, and when ENet and YOLO were used together, the average recognition error was 2.913. When more people were in the picture, more errors appeared when using YOLO without ENet. However, when ENet and YOLO were used together, errors were be reduced and accurate recognition was achieved.

### 4.3. Experimental Results: Memory Consumption

**Figure 16:**
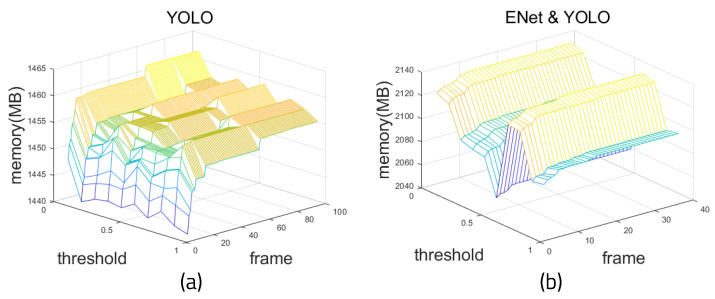
Relationship between memory usage and threshold: (**a**) YOLO and (**b**) ENet and YOLO.

Figure 16 shows the results of measuring the amount of memory used when running the algorithms on the Windows 10-based PC. Figure 16a shows the memory measurement of YOLO alone, and Figure 16b shows the memory usage of YOLO and ENet. When YOLO detects an object, it is judged as a matching object when the threshold is 0.5 or more through the IoU (Intersection Over Union) of the Bounding Box and the Correct Answer Box, which contain information about the predicted object. The higher the threshold, the more consistent the correct answer; it is important to set an appropriate threshold, because when the reference threshold is too high, the detection rate is lower [29]. It can be seen that minimal memory is used at a certain threshold. The reason for this is that when the threshold is too low, there are a lot of objects detected and consume a lot of memory. However, when the threshold is too high, no objects are detected, and the memory usage is low. When the threshold is 0.4, the average memory usage is 2.067 GB.

When using ENet and YOLO together, the memory usage is obviously higher than when using YOLO alone because the models for the two architectures must be loaded separately. Despite the fact that the number of memory bytes is increased, the error is greatly decreased. In addition, this method is valid because it can be used effectively on an embedded board.

### 4.4. Experimental Results: Background Complexity

**Figure 17:**
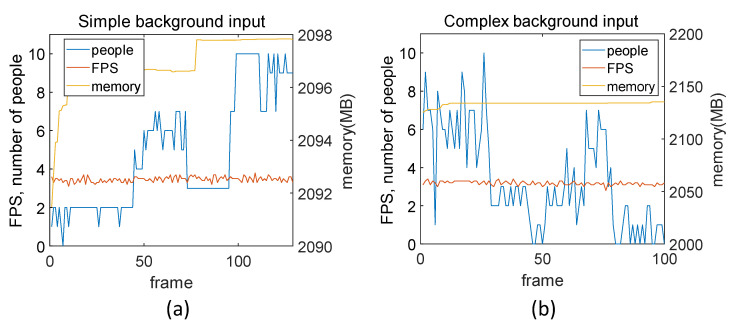
Time measurement result of complexity of background: (**a**) simple background and (**b**) complex background.

Figure 17 shows how the complexity of the background in the picture affects the calculation when executed on the Windows 10-based PC. The background was considered to be complicated if the variance was large by calculating the average of the RGB values and the resulting deviation and variance in the background aside from the recognized person. First, photos with complex backgrounds and people on a white background were visually selected and entered into the webcam in real time. These were then measured by dividing the complex background and the simple background into twenty photos each.

**Figure 18:**
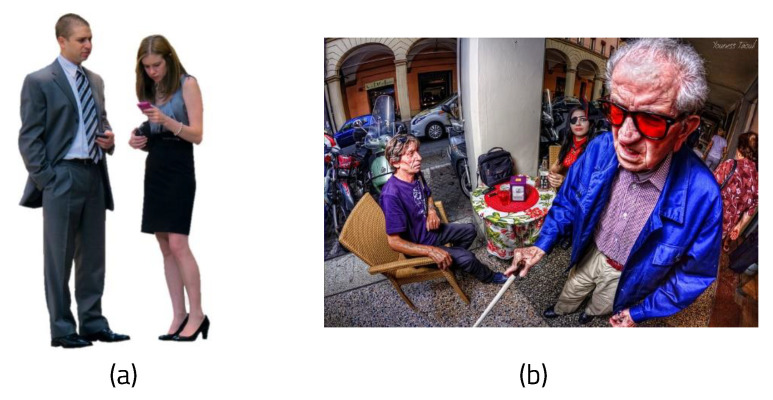
Example image of complexity of background: (**a**) simple background and (**b**) complex background.

Figure 18 shows example images of background complexity. The variance of the RGB values is 0.047 in Figure 18a and 0.132 in Figure 18b. When the background is simple, the average FPS is 3.46, the average memory is 2096.937 MB, and the average variance of the RGB values is 0.0655. When the background is complex, the average FPS is 3.17, the average memory is 2133.625 MB, and the average variance of RGB is 0.177. Because the difference in the RGB average variance is large, the complexity of the background can be distinguished by the variance value. When performing semantic segmentation using ENet, it can be seen that it is not necessary to exclude the background of the photos. Because the background is simple, segmentation requires less time and less memory.

### 4.5. Experimental Results: Performance on Boards

**Figure 19:**
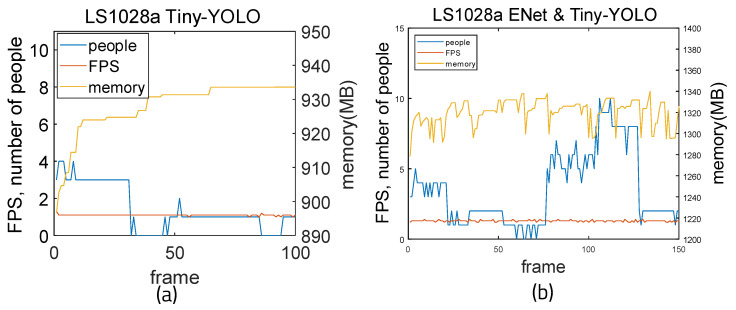
Measurement results for LS1028a board: (**a**) Tiny-YOLO and (**b**) ENet and Tiny-YOLO.

Figure 19 shows the measurement results when running on the LS1028a board. The Tiny-YOLO weights [30] were used because light weights should be used on the board. Tiny-YOLO is lighter than YOLO; while it has lower accuracy, it is more suitable because of its small size. When using Tiny-YOLO alone, the average FPS is 1.1 and the average memory usage is 930.08 MB. Calculating the accuracy using the previous method, the average accuracy is 0.2569 and the average error is 1.88. The precision is 0.997, recall is 0.539 and F1-score is 0.699. When using ENet and Tiny-YOLO together, the average FPS is 1.3 and the average memory usage is 1320.08 MB. The mean accuracy is 0.5866 and the mean error is 1.52. Precision is 0.968, recall is 0.711, and F1-score is 0.828. It can be seen that FPS, accuracy, and F1-score all improved when ENet was integrated.

**Figure 20:**
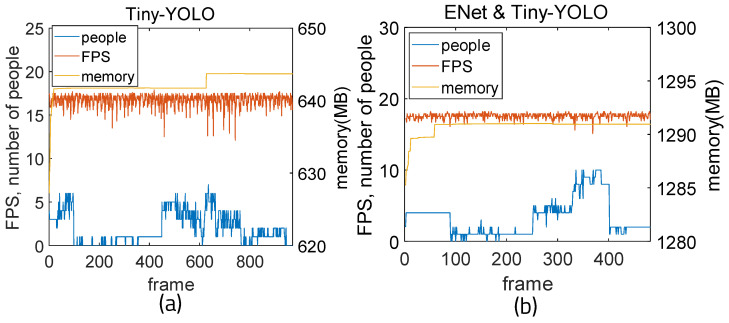
Time measurement results for Tiny-YOLO: (**a**) Tiny-YOLO and (**b**) ENet and Tiny-YOLO.

FPS was reduced when running on a much lighter board than a PC. Using Tiny-YOLO weights, the amount of memory is reduced by almost half; however, it can be seen that there is a big difference in accuracy between the two algorithms. When more weights with low accuracy are used, the accuracy is increased by setting the ROI using ENet. For comparison, in Figure 20, the Windows-based PC was measured using Tiny-YOLO weights. When using YOLO-Tiny and ENet, the average fps is 17.57 and the average memory usage is 1290.75 MB. The mean accuracy is 0.544 and the mean error is 1.46. Precision is 0.997, recall is 0.709, and F1-score is 0.828. When using Tiny-YOLO alone, the average fps is 16.72 and the average memory usage is 642.33 MB. The mean accuracy is 0.263 and the mean error is 3.44. Precision is 0.977, recall is 0.389, and F1-score is 0.556. It can be seen that Tiny-YOLO uses less memory and has higher fps than using YOLO alone, while when ENet is integrated, the accuracy and fps are enhanced and the F1-score increases.

**Figure 21:**
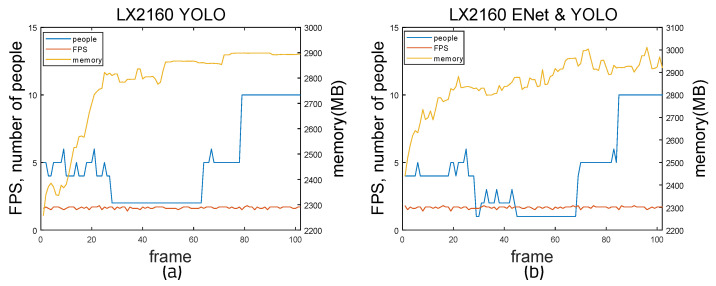
Time measurement result of LX2160 board: (**a**) YOLO and (**b**) ENet and YOLO.

**Figure 22:**
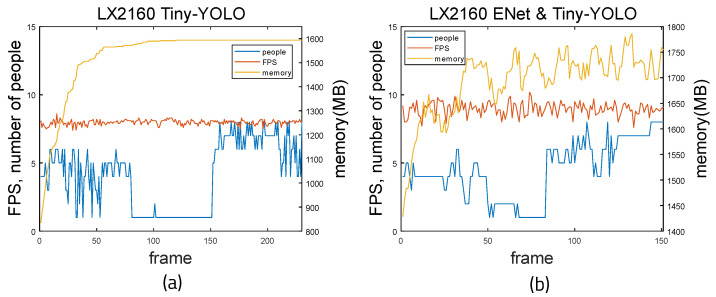
Time measurement result of Tiny-YOLO weight on LX2160 board: (**a**) Tiny-YOLO and (**b**) ENet and Tiny-YOLO.

Figure 21 and Figure 22 show the measurement results when running on the LX2160a board. Both YOLO weights and Tiny-YOLO weights were used, as this board has more memory than the LS1028a board. When using YOLO alone, the average FPS is 1.643 and the average memory usage is 2775.02 MB. By calculating the accuracy using the previous method, the average accuracy is 0.13 and the average error is 1.72. Precision is 0.937, recall is 0.768, and F1-score is 0.844. When using ENet and YOLO together, the average FPS is 1.664 and the average memory usage is 2855.165 MB. The mean accuracy is 0.1553 and the mean error is 1.25. Precision is 0.973, recall is 0.788, and F1-score is 0.87. When using Tiny-YOLO alone, the average FPS is 7.967 and the average memory usage is 1527.21 MB. The average accuracy is 0.178 and the average error is 2.6. Precision is 0.88, recall is 0.629, and F1-score is 0.87. When using ENet and Tiny-YOLO together, the average FPS is 8.96 and the average memory usage is 1692.72 MB. The mean accuracy is 0.157 and the mean error is 2.42. Precision is 0.943, recall is 0.662, and F1-score is 0.777. It can be seen that when using ENet and YOLO together, fps is higher and error is lower. Furthermore, the difference in memory usage is small at 100 MB. When using ENet and Tiny-YOLO, fps values are much higher than for YOLO alone. The gap between memory usage of ENet with Tiny-YOLO and Tiny-YOLO alone is small.

### 4.6. Experimental Results: Power Consumption

**Figure 23:**
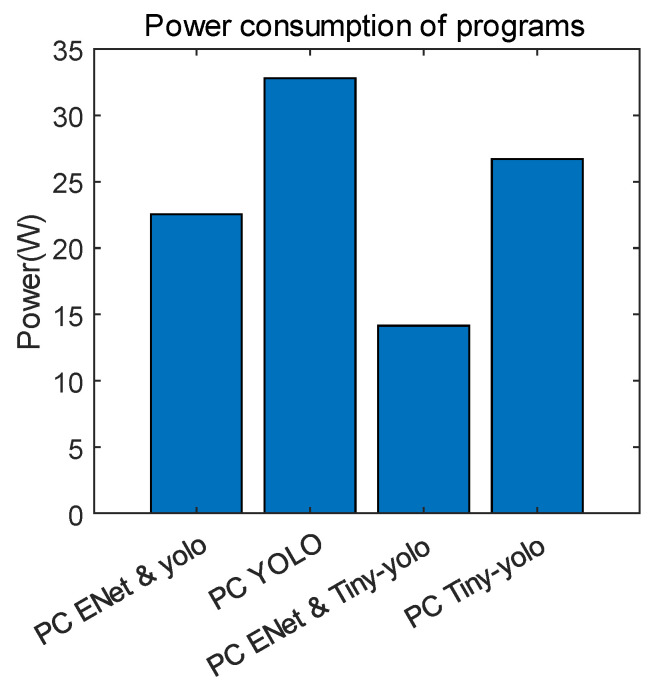
Power consumption results.

Figure 23 illustrates the power consumption of each algorithm. They were executed on the host PC, and the threshold was fixed to 0.4. The power consumption of the architectures was determined running on an AMD Ryzen 7 3800XT 8-Core Processor with 3.90 GHz, RAM 32.0 GB, and Windows 10 Pro. The power consumption results are the average value of the total CPU power used by the architecture when only the CPU is used. The amount of power used by the architecture to process the same data was measured by putting the using picture as the input for the same period of time. It was not measured on the boards, because it was only necessary to compare the validity of the architectures’ power usage. When using ENet and YOLO, the power consumption is 22.54 W, while when using YOLO alone is 32.831 W. Executing ENet and Tiny-YOLO consumed 14.14 W, while Tiny-YOLO alone consumed 26.716 W. It can be seen that using ENet with YOLO reduces power consumption by approximately 10 W, which is useful for lightweighted embedded boards, as Tiny-YOLO uses less power than YOLO.

### 4.7. Experiments in Different Deep Learning Frameworks

**Figure 24:**
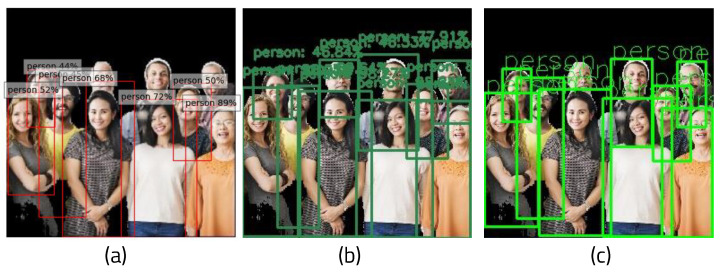
Results for different frameworks: (**a**) SSD, (**b**) MobileNet, and (**c**) Faster R-CNN.

**Figure 25:**
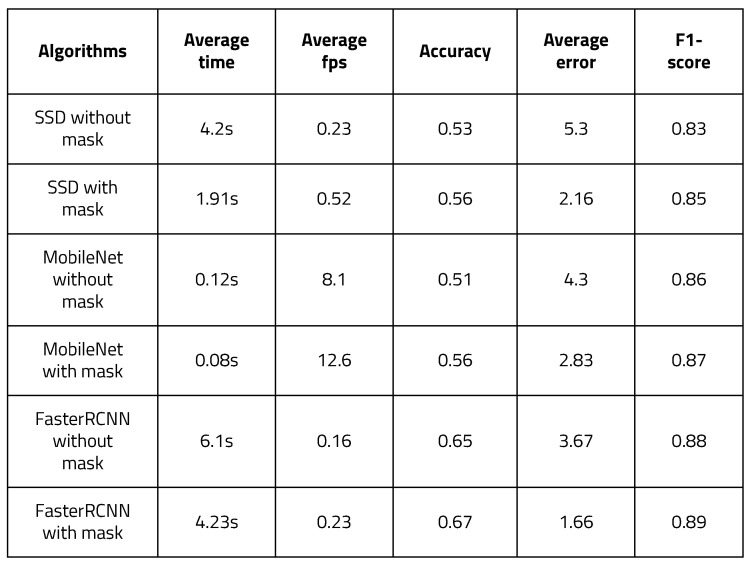
Time and accuracy measurement of different frameworks.

To evaluate the effects of the architecture, it was evaluated with various deep learning frameworks. Figure 24 shows images resulting from the different frameworks. As there are two types of state-of-the-art target detection algorithms based on CNN, SSD and Faster R-CNN are assessed. SSD is a one-stage end-to-end target detection algorithm. Faster R-CNN is a two-stage target detection algorithm, in which the process is divided into two phases. A lightweighted architecture, MobileNet, is tested as well, as it can be performed efficiently on the boards. Figure 25 illustrates the time and accuracy measurement of these frameworks. The results when executing the algorithm using YOLO on the host PC and the FPGA board were similar in terms of the trends in the two environments; thus, the experiment was conducted only on the host PC. The time and FPS variance between the masked input image and the image without masking was 2.1 times faster with SSD, 1.5 times with MobileNet, and 1.4 times with Faster R-CNN. There was not much difference in the accuracy or F1-scores of the frameworks. However, the average error fell by about half in each framework. Judging from these results, it can be concluded that the proposed algorithm is efficient for object detection processing.

**Figure 26:**
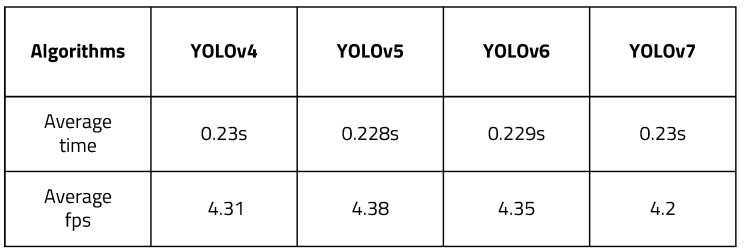
Time and FPS measurement of different versions of YOLO.

There are several versions of YOLO, which has been developed up to version 7 as of 2022. YOLOv4, v5, v6, and v7 were tested with the proposed algorithm. YOLOv4 [31] increased AP (Average Precision) and FPS by 10% and 12%, respectively, compared to v3. In v4, various deep learning techniques (WRC, CSP etc.) are used to improve performance, and the CSPNet-based backbone (CSPDarkNet53) is used. YOLOv5 [32] uses the same CSPNet-based backbone as YOLOv4. It is a PyTorch implementation, not Darknet, which is different from previous versions. Compared to v4, YOLOv5 is characterized by being able to configure and implement the environment more easily. YOLO v6 [33] is slightly harder to use in practice than YOLOv5, and there are not as many established paths and articles on how to actually use networks for training, deployment, and debugging. Starting with YOLOv6, it is possible to detect objects of various sizes, with the existing three scales increased to four. YOLOv7 [34] proposes a trainable bag-of-freebies method for real-time object detection that can significantly improve detection accuracy without increasing inference cost. In addition, it uses ’extend’ and ’compound scaling’ methods for real-time object detectors that can effectively utilize parameters and computation. We tested the models on an LS1028a board as an example of a lightweight embedded board. Figure 26 shows the time and fps measurement result with different versions of YOLO. YOLOv4 took 0.23 s and had 4.31 FPS. YOLOv5 took 0.228 s and had 4.38 FPS. YOLOv6 took 0.229 s and had 4.35 FPS. YOLOv7 took 0.23 s and had 4.2 FPS. The higher versions had better average time and fps. In addition, it can be seen that all versions above YOLOv3 showed improved results compared to the previous experiment.

## 5. Conclusions and Discussion

This paper introduces an ROI masking method using semantic segmentation with ENet and an algorithm that can execute object recognition in real time on a lightweight embedded board using YOLO. It uses a webcam to receive real-time image input. Using an ENet model that has been trained to recognize only humans, image frames are converted to an appropriate size and then segmented. After binarizing the segmentation result and masking it for the ROI, the resulting images are used for object recognition with YOLO.

Our results show that using ENet to set the ROI improves accuracy significantly. The number of errors drops from 7 to 2.9. This algorithm can be judged valid because this increase in accuracy is achieved while increasing memory usage by only about two times, while power consumption is reduced from 32.8 W to 22.54 W when using ENet as the ROI setting. By testing the algorithm in several deep learning framework such as SSD, MobileNet, and Faster RCNN, we found that the average time required decreased by about 1.5 times, and the number of errors diminished to half. As a result of testing the different versions of YOLO developed thus far, the results for version 5 were the fastest at 4.38 FPS. Filtering the input image once using segmentation and then using the result to recognize an object increases the accuracy and reduces the required amount of computation. In addition, by dividing the code into two operations, the amount of computation can be further reduced. If the divided code is shared on different embedded boards to process images while communicating with each other, a lighter real-time image processing algorithm can be implemented.

Through this study, it has been found that the efficiency of object recognition can be greatly increased by using two deep learning models. It is expected that this method can be used for autonomous driving and IoT, which are fields that currently need to recognize people using object recognition. In addition, because it can be implemented on a much lighter and cheaper boards than the boards currently used in object recognition, it can be seen that the potential for grafting is high. Experimenting with different deep learning object recognition frameworks and different versions shows that this algorithm can be implemented flexibly. Therefore, we predict that the method devised here can be used with several deep learning and machine learning-based object recognition structures currently being studied.

In the future, research can be conducted into real-time object recognition based on deep learning to improve the accuracy in various environments and to optimize for operation on even lighter embedded boards. In addition, research on reducing the overflow that occurs during real-time image analysis by utilizing communication technologies such as socket communication can be studied. In addition, because the current object recognition deep learning algorithm was developed very rapidly in several ways, we plan to study it further in order to execute it more flexibly in various frameworks and languages according to the flow.

## Data Availability

Not applicable.

## Abbreviations

The following abbreviations are used in this manuscript:

YOLO	You Look Only Once
ROI	Region Of Interest
ENet	Efficient Neural network
R-CNN	Regions with Convolutional Neuron Networks
FCN	Fully Convolutional Networks
CNN	Convolutional Neural Network
OpenCV	Open-Source Computer Vision
FPS	Frames Per Second
